# The neonatal ketone body is important for primordial follicle pool formation and regulates ovarian ageing in mice

**DOI:** 10.1093/lifemeta/loac017

**Published:** 2022-08-11

**Authors:** Xin-Ying Wang, Xin-Ge Zhang, Yong-Juan Sang, Dan-Yang Chong, Xiao-Qiang Sheng, Hai-Quan Wang, Chao-Fan Yang, GuiJun Yan, Hai-Xiang Sun, Chao-Jun Li

**Affiliations:** Ministry of Education Key Laboratory of Model Animal for Disease Study, Model Animal Research Center of Nanjing University, Nanjing, Jiangsu 210093, China; State Key Laboratory of Reproductive Medicine and China International Joint Research Center on Environment and Human Health, Center for Global Health, School of Public Health, Nanjing Medical University, Nanjing, Jiangsu 211166, China; Ministry of Education Key Laboratory of Model Animal for Disease Study, Model Animal Research Center of Nanjing University, Nanjing, Jiangsu 210093, China; State Key Laboratory of Reproductive Medicine and China International Joint Research Center on Environment and Human Health, Center for Global Health, School of Public Health, Nanjing Medical University, Nanjing, Jiangsu 211166, China; Center for Reproductive Medicine, Department of Obstetrics and Gynecology, Nanjing Drum Tower Hospital, the Affiliated Hospital of Nanjing University Medical School, Nanjing University, Nanjing, Jiangsu 210093, China; Center for Reproductive Medicine, Department of Obstetrics and Gynecology, Nanjing Drum Tower Hospital, the Affiliated Hospital of Nanjing University Medical School, Nanjing University, Nanjing, Jiangsu 210093, China; Ministry of Education Key Laboratory of Model Animal for Disease Study, Model Animal Research Center of Nanjing University, Nanjing, Jiangsu 210093, China; State Key Laboratory of Reproductive Medicine and China International Joint Research Center on Environment and Human Health, Center for Global Health, School of Public Health, Nanjing Medical University, Nanjing, Jiangsu 211166, China; Center for Reproductive Medicine, Department of Obstetrics and Gynecology, Nanjing Drum Tower Hospital, the Affiliated Hospital of Nanjing University Medical School, Nanjing University, Nanjing, Jiangsu 210093, China; State Key Laboratory of Reproductive Medicine and China International Joint Research Center on Environment and Human Health, Center for Global Health, School of Public Health, Nanjing Medical University, Nanjing, Jiangsu 211166, China; Center for Reproductive Medicine, Department of Obstetrics and Gynecology, Nanjing Drum Tower Hospital, the Affiliated Hospital of Nanjing University Medical School, Nanjing University, Nanjing, Jiangsu 210093, China; State Key Laboratory of Reproductive Medicine and China International Joint Research Center on Environment and Human Health, Center for Global Health, School of Public Health, Nanjing Medical University, Nanjing, Jiangsu 211166, China; Center for Reproductive Medicine, Department of Obstetrics and Gynecology, Nanjing Drum Tower Hospital, the Affiliated Hospital of Nanjing University Medical School, Nanjing University, Nanjing, Jiangsu 210093, China

**Keywords:** ketone body, primordial follicle pool, Hmgcs2, ROS, POA

## Abstract

Adverse nutritional conditions during the perinatal stage are related to early menopause in adulthood; however, the underlying mechanism is still unclear. Herein, we revealed that colostrum-activated ketone body elevation during the postnatal stage regulated primordial follicle reservoir size and then affected ovarian ageing. We found that the expression of the ketogenesis rate-limiting enzyme 3-hydroxy-3-methylglutaryl-CoA synthase 2 (Hmgcs2) was largely enhanced during primordial follicle pool formation after birth and might be activated in the ovaries by colostrum. Reactive oxygen species (ROS) elevation in the ovaries leads to follicle apoptosis to deplete damaged follicles, while Hmgcs2 deficiency enhances follicle apoptosis and thus decreases the size of the primordial follicle pool and leads to premature ovarian ageing (POA), which might be related to the activation of cellular endogenous antioxidant system. All these defects could be rescued by ketone body administration, which suppressed ROS-activated follicle apoptosis. Our results suggest that the internal metabolic homeostasis of newborn mice is critical for the primordial reservoir and that any intrauterine and perinatal undernutrition could result in POA.

## Introduction

Premature ovarian ageing (POA) refers to an early decline in ovarian function; it is the main cause of infertility in older women and is characterized by a markedly reduced ovarian reservoir [1]. The ovarian reservoir is dependent on the size of the primordial follicle pool that is established during the perinatal stage. Oocyte DNA damage emerges during early meiosis in primordial-stage follicles, which triggers follicular apoptosis and follicle depletion during the perinatal stage [2]. Thus, the total number of primordial follicles is stabilized soon after birth. Various abnormal situations occur in the ovaries or the whole body, which cause reserve inefficiency, even result in POA [3, 4].

In addition to the intracellular insults during primordial germ cell mitosis and meiosis, there are also dramatic changes in the extracellular environment. When a baby is born, the acquisition of dietary lipids from colostrum and access to more oxygen by spontaneous breathing reprogram the metabolic pattern of the body, namely, anaerobic glycolysis switches to aerobic fatty acid (FA) β-oxidation in the mitochondria. Thus, newborns are more susceptible to oxidative stress than adults. The physiological level of reactive oxygen species (ROS) is beneficial during folliculogenesis, oocyte maturation, and embryogenesis [5]. The generation of a moderate level of ROS (i.e. 60–80 ng/oocyte) may trigger meiotic resumption after diplotene arrest [6]. However, a high level (>80 ng/oocyte) of ROS can accelerate oocyte ageing, decrease oocyte quality, and induce oocyte apoptosis, thus causing a variety of ovarian diseases, including POA [7, 8]. Eva’s team found that DNA damage response processes act across the life course to govern human ovarian ageing, such as shaping the ovarian reserve and its rate of depletion [9]. Therefore, the ROS level in the ovaries during the neonatal stage should be carefully controlled to protect against excessive DNA damage and oocyte apoptosis [10].

When newborns intake abundant dietary lipids from colostrum, the sudden increase in FAs largely enhances β-oxidation in the mitochondria [11]. The extra acetyl-CoA derived from β-oxidation is shuttled into ketogenesis regulated by the rate-limiting enzyme 3-hydroxy-3-methylglutaryl-CoA synthase 2 (Hmgcs2) [12]. Ketone bodies are not only an important alternative energy source for almost all organs [13, 14], but also direct regulators of enzyme activity and participants in intracellular signal transduction [15]. It has been reported that *Hmgcs2* deletion and ketogenesis deficiency in neonatal mice lead to mitochondrial protein hyperacetylation and result in hepatic mitochondrial dysfunction and lipid accumulation [16]. Furthermore, a single-cell sequencing report showed that the ketogenic gene *Hmgcs2* remains at a high level in pregranulosa cells of the ovaries in mice during the perinatal period [17]. However, the relationship between these two simultaneous events, ketone body increase and primordial follicle reservoir formation, has not been clearly explored.

It was previously shown that metabolic disorders, such as diabetes and obesity, are related to compromised human reproductive function [18]. An interesting review summarized that women born in famine years have a significantly earlier menopausal age, which indicates the importance of early nutrition during the newborn stage [19]. These previous studies inspired us to determine the relationship between early ketone body production and primordial follicle reservoir formation.

## Results

### Hmgcs2 and ketone body levels are significantly increased in the ovaries of neonatal mice

**Figure 1:**
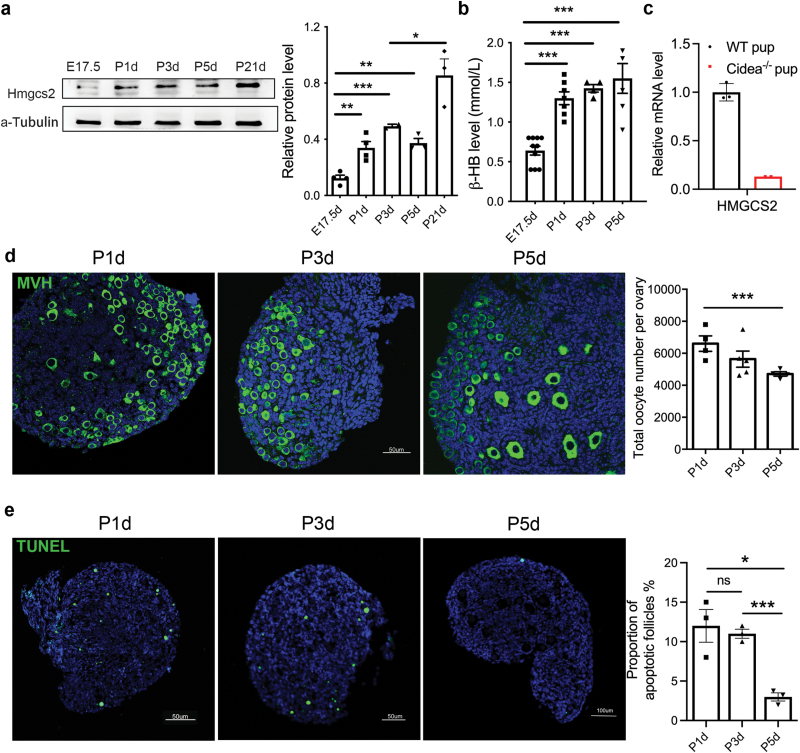
Primordial follicle reserve and the ketone body production. (a) Western blot analysis of the *Hmgcs2* expression in E17.5d, P1d, P3d, P5d, and P21d ovaries (*n* = 3−4). (b) The concentration of serum ketone bodies in neonatal mice (*n* = 4−10). (c) The relative mRNA level of *Hmgcs2* in the ovaries of Cidea^−/−^ pups (*n* = 3). (d) Immunofluorescence staining of the wild-type neonatal mouse ovaries. Green fluorescence indicates MVH, and blue fluorescence indicates DAPI staining (*n* = 3−5). (e) Follicle apoptosis in wild-type neonatal mice. Green fluorescence indicates positive TUNEL staining, and blue fluorescence indicates DAPI staining (*n* = 3). Data are shown as means ± s.e.m. *P < 0.05, **P < 0.01, ***P < 0.001, ns, no significance.

We detected the expression pattern of *Hmgcs2* in ovaries from embryonic 17.5-day (E17.5d), postnatal 1-day (P1d), 3-day (P3d), 5-day mice (P3d), and 21-day mice (P21d), and found that the Hmgcs2 expression level was largely enhanced in the neonatal ovaries during the period of primordial follicle pool formation (Fig. 1a). In addition, Hmgcs2 protein was even significantly increased in the ovary of P21d mice compared with that in P3d mice, which might be due to the increased proportion of granulosa cells after follicle development (Fig. 1a). Serum ketone bodies, the catalytic products of Hmgcs2, were also significantly increased at concentrations above 1.0 mmol/L after birth (Fig. 1b), which is comparable to the levels observed under pathological conditions such as overnight fasting [20]. Cidea is an important transcriptional coactivator that regulates mammary gland milk lipid secretion [21]. We detected the mRNA level of *Hmgcs2* in P1.5d pups generated by crossing Cidea^−/−^ females and wild-type males, and found that there was no *Hmgcs2* expression in the ovarian tissue of Cidea^−/−^ pups (Fig. 1c), indicating that colostrum is critical for the induction of *Hmgcs2* expression and ketone body production in the ovaries. In the period of primordial follicle pool formation, the total follicle number decreased with postnatal day (Fig. 1d). Examination of apoptosis in the ovaries also showed that the number of apoptotic oocytes was high in P1d and P3d but decreased in P5d ovaries, as primordial follicle pool formation was finished before P5d (Fig. 1e). Therefore, our data indicated that the neonatal nutritional status and *Hmgcs2-*regulated ketone bodies might be related with the formation of primordial follicle pool.

### *Hmgcs2* deficiency leads to POA

**Figure 2:**
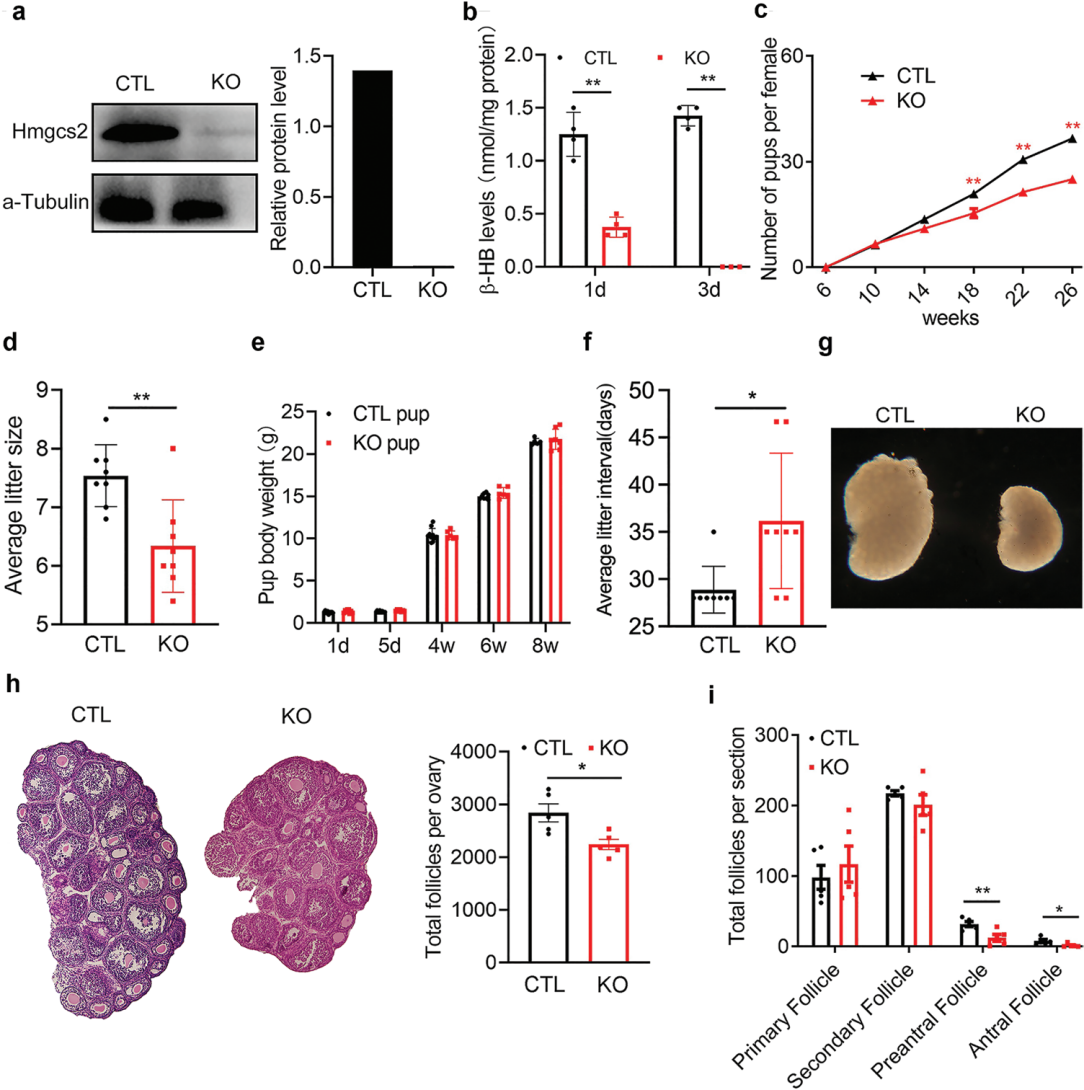
*Hmgcs2* deficiency leading to subfertility. (a) Western blot analysis used to detect the knockdown efficiency in *Hmgcs2*-null mice. (b) The serum β-HB level (*n* = 4). (c) Cumulative fertility test of mice in the KO group and the CTL group (*n* = 6). (d) The litter sizes of the KO group and the CTL group (*n* = 6). (e) The weight of pups born to mice in the KO group and CTL group (*n* = 6). (f) Litter intervals of the KO group and the CTL group (*n* = 6). (g) The shape of the ovaries in the 21-day KO group and CTL group. (h) Total number of ovarian follicles in the 21-day KO group and CTL group (*n* = 5). (i) The number of preantral follicles and antral follicles in the 21-day KO group and CTL group (*n* = 5). Data are shown as means ± s.e.m. *P < 0.05, **P < 0.01.

To explore the function of ketone bodies in primordial follicle reservoir formation in mice, we constructed systemic *Hmgcs2* knockout (KO) mice by crossing *Hmgcs2-*floxed mice with EIIA-Cre transgenic mice. Western blotting showed that the Hmgcs2 protein level was almost undetectable in the ovaries of the KO mice (Fig. 2a), and that the ketone body level was decreased significantly (Fig. 2b), which indicated that Hmgcs2 was successfully knocked out in these mice. We found that there was no difference in fertility between young KO females and wild-type females until 18-week-old (Fig. 2c). Litter size (Fig. 2d) and pup weight (Fig. 2e) were not affected by the systemic absence of ketone bodies in young mice. With increasing age, the litter size of KO mice gradually decreased (Fig. 2d), and the litter interval was significantly prolonged (Fig. 2f). In addition, 6-month-old Hmgcs2-KO mice showed an incomplete oestrous cycle, in which the oestrous frequency decreased and the cycle disordered (Supplementary Fig. S1a and b). Examination of the ovaries suggested that the fertility decrease might be due to defects in ovarian development, as the ovarian size was significantly reduced for the ovaries of 3-week-old KO mice (Fig. 2g). Haematoxylin and eosin (HE) staining showed that the total number of follicles was also decreased in the KO group (Fig. 2h), while primary and secondary follicles were not affected (Fig. 2i). This indicated that the ovarian follicle reservoir in *Hmgcs2-*deficient mice was decreased, while the recruitment/activation of primordial follicles was normal. All the data suggested that *Hmgcs2* might be related to the maintenance of adulthood ovarian function through regulation of ovarian follicle pool formation during the neonatal stage. However, how this occurs was unclear.

### *Hmgcs2* deficiency does not affect cell lineage development in postnatal ovary

**Figure 3:**
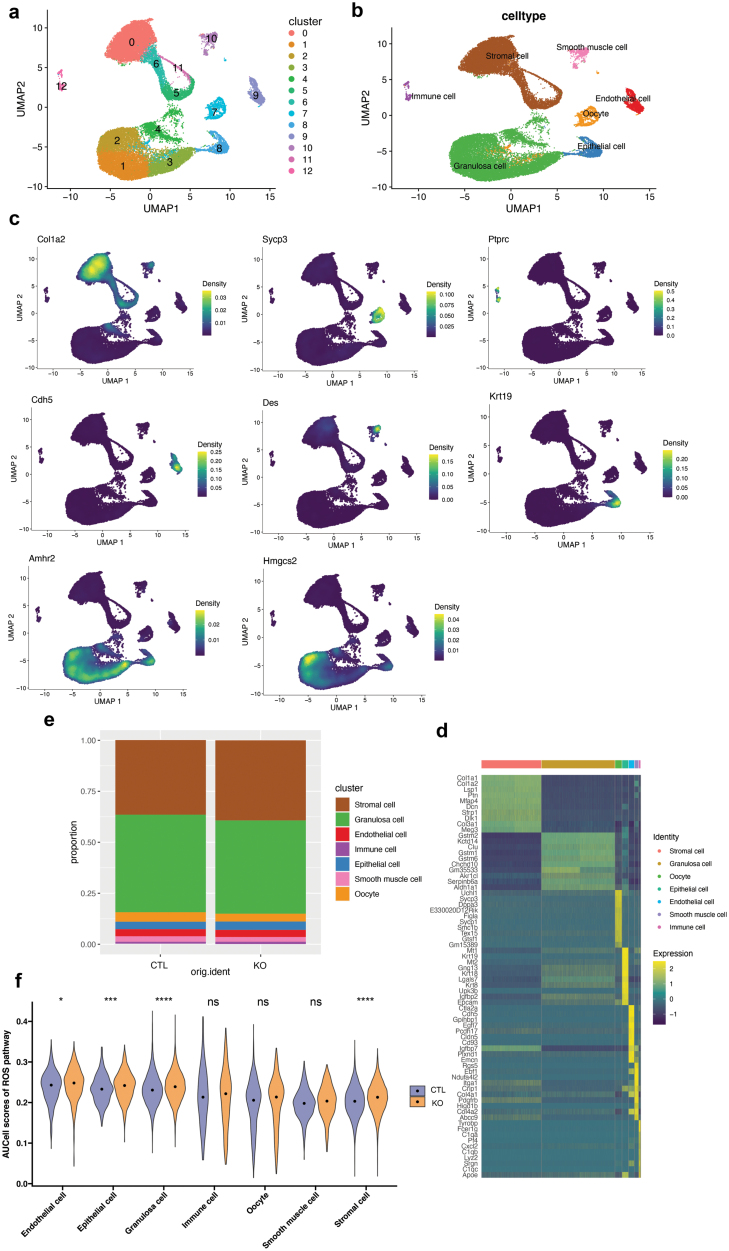
Distinct ovarian cell subpopulations with transcriptional signatures determined by single-cell RNA-sequence analysis of P3d ovaries. (a) UMAP plot showing 12 clusters of P3d ovaries. (b) UMAP plot showing 7 cell types of P3d ovaries. (c) UMAP plots characterizing representative transcriptional regulators for different cell types. (d) Heatmap showing expression signatures of top 10 specifically expressed genes in each cell type. (e) The proportion of seven cell types in P3d ovaries. (f) The AUC score of ROS pathways in seven cell types in P3d ovaries. *P < 0.05, ***P < 0.001, ****P < 0.0001, ns, no significance (two-tailed *t*-test).

To reveal the intrinsic reason for ovarian follicle pool formation defect after *Hmgcs2* deletion, we first did single-cell RNA sequencing of P3d ovary to compare the cell lineage development of KO and control (CTL) mice. At first, we classified the cells in P3d ovary to 12 clusters using Uniform Manifold Approximation and Projection (UMAP) method (Fig. 3a). According to the gene-expression profiles of cell-type-specific markers, these cell clusters in the UMAP plot were classified into seven cell subtypes, including oocyte, stromal cell, granulosa cell, immune cell, smooth muscle cell, endothelial cell, and epithelial cell (Fig. 3b−d). For example, stromal cell marker, Col1a1, was specifically expressed in clusters 0, 6, 5, and 11, thus these four clusters belong to stromal cells [22]. *Sycp3* is well-known germline marker gene which specifically expressed in cluster 7 (oocyte) [22]. *Amhr2*, the granulosa marker, was found in clusters 1, 2, 3, and 4, which also specifically expressed *Hmgcs2* [22] (Fig. 3b−d). But when we counted the number of different subtypes, we found that there was only a little difference in subtypes proportion between the KO and CTL groups, which means that *Hmgcs2* deletion does not affect the cell lineage development in the process of primordial follicle pool formation (Fig. 3e). Since ROS play an important role in regulating early follicle development and apoptosis [10], we calculated the scores of ROS-related genes obtained from hallmark category in Molecular Signatures Database (v7.5.1) using AUCell package (Fig. 3f). We found that the activation level of ROS pathway in the KO group was higher in endothelial cells, epithelial cells, granulosa cells, and stromal cells than that of the CTL group (Fig. 3f). While this difference was statistically significant, the ROS levels were shown elevated in both Hmgcs2-deficient and wild-type neonatal mice under physiological conditions upon stimulation of autonomous respiration [23]. Therefore, we further clarified the effect of higher ROS in the KO group on the formation of primordial follicle pool in the following studies.

### *Hmgcs2* in granulosa cells does not affect primordial follicle pool formation

**Figure 4:**
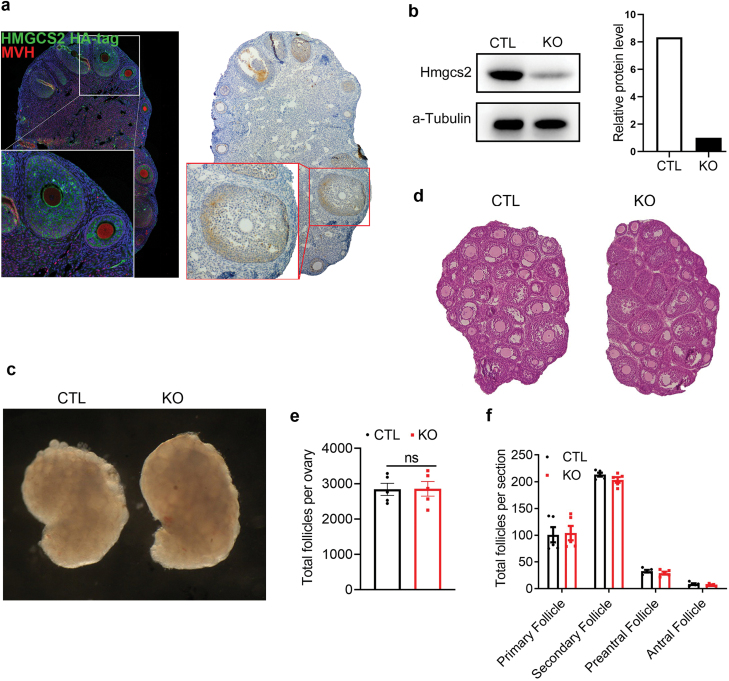
*Hmgcs2* KO in granulosa cells does not affect primordial follicle pool formation in mice. (a) Immunofluorescence staining and immunohistochemical staining of 8-week-old Hmgcs2 HA-tag mice ovary; green fluorescence indicates HA-tag, and blue fluorescence indicates DAPI staining. (b) Western blot analysis used to detect the efficiency of granulosa cell-specific knockdown of Hmgcs2. (c) The ovarian morphology of mice in the KO group and CTL group at 21-day-old. (d) HE staining of samples from mice in the CTL group and KO group at 21 days. (e) Total number of ovarian follicles in KO and CTL mice at 21 days (*n* = 5). (f) The number of follicles in each stage in the ovaries in the 21-day KO group and CTL group (*n* = 5). Data are shown as means ± s.e.m. ns, no significance.

According to our single-cell sequencing data and Allan’s group [17], we found that *Hmgcs2* was highly expressed in mouse ovarian granulosa cells but not in oocytes (Fig. 3c and d). We also confirmed it by immunofluorescence staining and immunohistochemical staining of 8-week-old Hmgcs2 HA-tag mice ovary (Fig. 4a). To distinguish the function of Hmgcs2 in mouse granulosa cells in primordial follicle pool formation, we knocked out *Hmgcs2* by crossing floxed mice with AmhR2-Cre mice (Fig. 4b). We found that there was no significant difference in ovarian morphology in 3-week-old mice between the granulosa cell-specific knockout *Hmgcs2* group and the CTL group (Fig. 4c). HE staining (Fig. 4d) showed that the total follicle number (Fig. 4e) and follicle development (Fig. 4f) were not affected by *Hmgcs2* granulosa cell-specific knockout. Our data suggested that the ovarian follicle reservoir is affected only by systemic *Hmgcs2* but not by the granulosa cell-specific function of *Hmgcs2*.

### The neonatal ketone body is critical for primordial follicle pool formation

**Figure 5:**
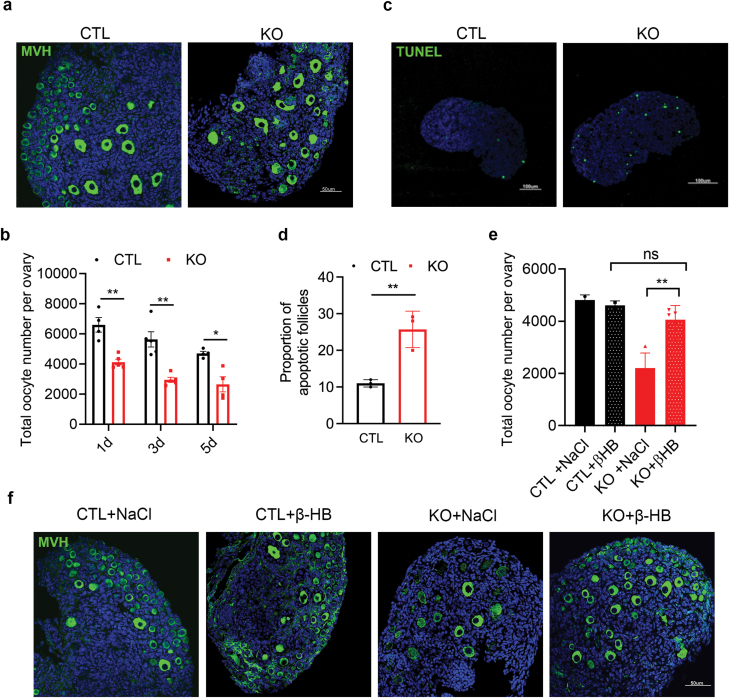
Defects in primordial follicle pool formation exhibited in the *Hmgcs2* KO mice. (a) Immunofluorescence staining results for P5d KO group and CTL group mice ovaries. Green fluorescence indicates MVH, and blue fluorescence indicates DAPI staining. (b) The number of oocytes in the KO group and CTL group mice ovaries at P1d, P3d, and P5d (*n* = 4). (c) Follicle apoptosis in KO group and CTL group mice ovaries at P3d. Green fluorescence represents positive TUNEL staining, and blue fluorescence indicates DAPI staining. (d) The proportion of apoptotic follicles in the P3d KO group and CTL group (*n* = 3). (e) The number of oocytes after supplying ketone bodies to mice in the KO group and the CTL group (*n* = 6). (f) Immunofluorescence staining results for four groups of mice at P5d. Green fluorescence indicates MVH, and blue fluorescence indicates DAPI. Data are shown as means ± s.e.m. *P < 0.05, **P < 0.01, ns, no significance.

We noticed that Hmgcs2*-*produced ketone body levels were significantly elevated during primordial follicle pool formation (Fig. 1b) and largely decreased by systemic *Hmgcs2* knockout (Fig. 2b). Thus, we examined the ovarian primordial follicle pool in P3d *Hmgcs2-*deficient mice. Compared with that in the CTL group, the number of oocytes in the KO group was significantly lower (Fig. 5a and b). What is the reason for the decrease in the primordial follicle pool with ketone body deficiency caused by *Hmgcs2* knockout? In the process of primordial follicle pool formation, a large number of follicles undergo apoptosis (Fig. 1e) to obtain better-quality follicles for subsequent normal development. Therefore, we speculated that more severe apoptosis of follicles leads to depletion of the primordial follicle pool in the context of ketone body deficiency. Therefore, we compared the apoptosis level in the ovaries of P3d mice between the CTL and *Hmgcs2* KO groups using a TUNEL assay. The results showed that the proportion of apoptotic follicles was largely increased in the KO group (Fig. 5c and d). To further confirm the function of ketone bodies in the primordial follicle pool, we administered β-Hydroxybutyrate (β-HB) on the second day after birth and examined the primordial follicle number in the ovaries of P5d mice. The results showed that ketone bodies could significantly alleviate the decrease in oocyte number caused by *Hmgcs2* knockout (Fig. 5e and f). Thus, our data indicated that the neonatal ketone body is critical for the formation of the follicle reservoir and functions by suppressing excess oocyte apoptosis during primordial follicle pool formation. Any reason for ketone body deficiency, such as undernutrition during the neonatal stage, might lead to the decline of the primordial follicle pool, and finally rise up to POA in adulthood.

### Neonatal ketone bodies inhibit excessive ROS elevation and primordial follicle apoptosis

**Figure 6:**
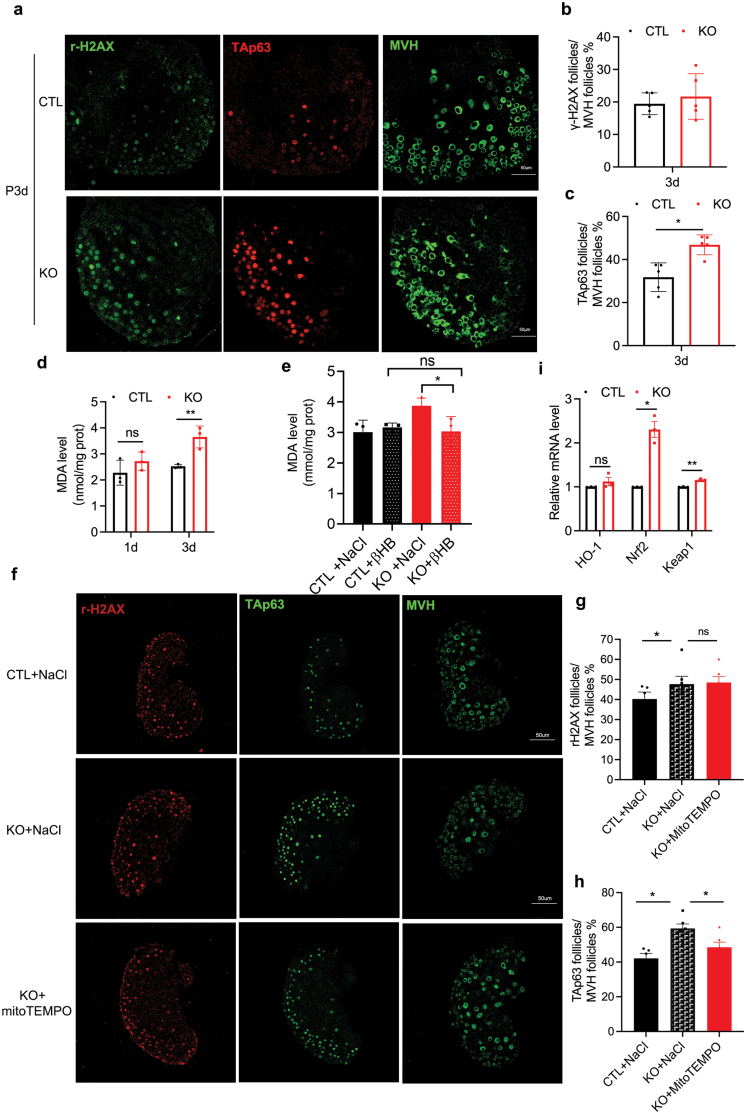
ROS elevation, DNA damage and activity of Nrf2/ARE antioxidant stress signalling pathway exhibited in the *Hmgcs2* KO mice. (a) Immunofluorescence staining of TAp63, γ-H2AX, and MVH in the KO group and CTL group at P3d. Red fluorescence indicates TAp63, the left green fluorescence indicates γ-H2AX in the same section, and the right green fluorescence indicates MVH staining in next section of TAp63 and γ-H2AX staining. (b) The ratio of γ-H2AX-positive follicle between P3d KO group mice and P3d CTL group mice (*n* = 5). (c) The ratio of TAp63-positive follicle in P3d KO group mice and the P3d CTL group mice. (d) The MDA levels of the P1d and P3d ovaries in the KO group and CTL group (*n* = 3). (e) The ROS levels in the KO group and the CTL group after supplying ketone bodies (*n *= 3). (f) Immunofluorescence staining of γ-H2AX, TAp63, and MVH in the three groups. Red fluorescence indicates γ-H2AX, the left green fluorescence indicates TAp63 in the same section, and the right green fluorescence indicates MVH staining in next section of TAp63 and γ-H2AX staining. (g) The ratio of γ-H2AX-positive follicle in the three groups (*n* = 6). (h) The ratio of TAp63-positive follicle in the three groups (*n* = 6). (i) The expression levels of Nrf2 and Keap1 in the P3d KO group and the CTL group (*n* = 3). Data are shown as means ± s.e.m. *P < 0.05, **P < 0.01, ns, no significance.

To figure out the reasons of apoptosis in neonatal ovary after *Hmgcs2* knockout, we double-stained TAp63 and γ-H2AX antibodies in the same section of ovary. To count the follicle number, we stained the continuous next section with MVH antibody. We found that the fluorescence intensity of the DNA double-strand break marker γ-H2AX was higher in P3d KO ovary, although the number of γ-H2AX-positive follicles has no significant difference in the P3d ovaries between KO group and CTL group (Fig. 6a and b). The proportion of TAp63-positive follicles in the P3d KO mice was also elevated (Fig. 6a and c). The higher γ-H2AX fluorescence intensity indicated that there were much more severe DNA double-strand breaked oocytes appeared in the process of primordial follicle formation in the KO mice ovary. Excess activated TAp63 also indicated that the apoptosis might be enhanced by *Hmgcs2* deletion. When we knocked out *Hmgcs2*, we found that the ROS level was significantly higher than that of wild-type mice on P3d, which was directly reflected by lipid perioxide, Malondialdehyde (MDA) concentration (Fig. 6d). The MDA result was consistent with the results of the ROS-pathway analysis in the single-cell sequencing (Fig. 3f). The elevation of ROS can be eliminated by intraperitoneal injection of β-HB (Fig. 6e). To prove the relationship among ROS, DNA damage and apoptosis, we treated neonatal KO mice with a mitochondrial antioxidant, mitoTEMPO, from P2d to P5d. The proportion of γ-H2AX-positive follicles was not significantly decreased in the KO mice ovary after being treated by MitoTEMPO (Fig. 6f and g), but the proportion of TAp63-positive follicles was largely decreased in the MitoTEMPO-treated KO ovary (Fig. 6f and h). The results indicated that mitochondrial Hmgcs2 deficiency resulted ROS is responsible for the primordial follicle apoptosis. The elevation of ROS in the KO mice ovary also activated the antioxidation response showed by the significantly enhanced erythroid-derived 2-related factor 2 (Nrf2) and Kelch-like ECH-associated protein 1 (Keap1) expression levels after *Hmgcs2* deletion (Fig. 6i). High-level ROS activated proapoptotic transcription factors, TAp63, and stimulated the activation of the Nrf2/ARE antioxidant stress signalling pathway in the ovaries of the *Hmgcs2* KO mice. All the above data indicated that a deficiency in neonatal ketone bodies would result in excessive elevation of ROS levels and thus induce apoptosis during primordial follicle formation.

**Figure 7:**
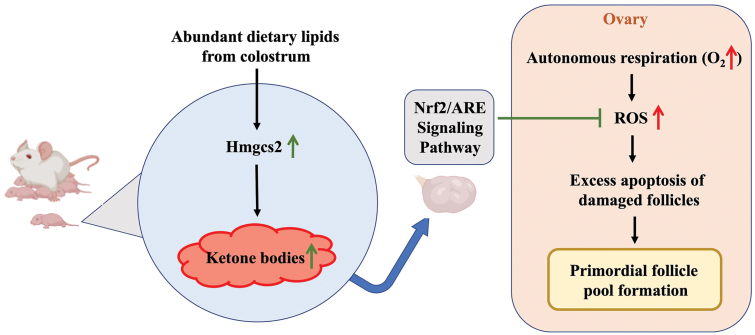
The working model for the function of ketone bodies in formation of primordial follicle pool. When the pup drink colostrum, the abundant dietary lipids activate the expression level of Hmgcs2, which can increase the level of systemic ketone bodies. Ketone bodies can regulate the Nrf2/ARE antioxidant stress signalling pathway, thus inhibit the production of excess ROS after autonomous respiration in the ovary. Otherwise, excess ROS would lead to excess apoptosis of follicle, resulting in the impairment of primordial follicle pool formation.

To summarize the function of ketone bodies in formation of primordial follicle pool, we proposed the following working model (Fig. 7). In the physiological process, maternal colostrum can activate the pup systemic ketone body elevation by increasing Hmgcs2 expression after birth. Ketone bodies can modulate the activity of Nrf2/ARE antioxidant stress signalling pathway to inhibit the accumulation of excess ROS that stimulated by autonomous respiration. Ketone bodies warrant the moderate apoptosis of follicles and thus ensure the quality and quantity of primordial follicle pool. In the ovary of ketone body-deficient mice, the excess ROS leads to the excess apoptosis of follicle, resulting in impairment of primordial follicle pool formation.

## Discussion

It was reported that foetuses which experience undernutrition in the intrauterine environment, e.g. those exposed to famine, suffer from early menopause [24]. Essentially, adverse foetal and infant conditions might influence follicle production in the first trimester, limiting the initial follicle pool or causing accelerated follicular loss thereafter [19]. Thus, early life could be an important period in determining follicular reserve and the age of natural menopause. However, the specific mechanism of this period is still unclear. In mice, some metabolic substrates and related hormones change dramatically after birth. Specifically, breast milk contains rich colostrum that can activate some signalling pathways to increase the transcription of metabolism-related genes, such as ketogenic genes [25]. It has been shown that the expression of the ketogenic gene *Hmgcs2* is maintained at a high level in bipotential pregranulosa cells and shows a rising trend in epithelial pregranulosa cells from E12.5d to P5d [17]. Herein, our study indicated that the ketone body, catalysed by Hmgcs2, might be an important component in regulating the formation of primordial follicles by suppressing ROS-induced follicle apoptosis after birth.

Although *Hmgcs2* is mainly expressed in the adult liver and produces ketone bodies from acetyl-CoA for extrahepatic organs [12], we found that its expression is increased in almost all organs of new-born mice, including the ovaries (Fig. 1a). Furthermore, the ketone body levels in the blood and ovaries of new-born mice are high (Fig. 1b), but largely reduced in the *Hmgcs2*-deficient mice (Fig. 2b). A high ketone body level is critical for the formation of the primordial follicle pool. The ROS levels in the body are transiently elevated in pregnant women [26]. There is a balance between an elevated-ROS maternal environment and high-level ketone bodies in neonatal mice. A suitable level of ROS induces moderate DNA damage and suitable apoptosis of primordial follicles [9], which helps strengthen the quality of the primordial follicle pool. In the ovaries of the *Hmgcs2* KO mice, ROS levels are largely elevated (Fig. 6d), which induces hyperapoptosis in normal follicles (Figs. 5c, 6a, and 6h). Thus, the *Hmgcs2* KO female mice have a smaller primordial reservoir size and show a POA phenotype, in which litter size is decreased and the litter interval is prolonged with age (Fig. 2d and f). Thus, we found that perinatal ketone body production is helpful for ameliorating excessive apoptosis during primordial follicle pool formation (Figs. 5e, 5f and 6e).

β-Hydroxybutyrate, one of the ketone bodies, has been shown to reduce the production of ROS, improving mitochondrial respiration. β-HB is an endogenous, specific inhibitor of class I histone deacetylases. When exogenous β-HB is administered, global histone acetylation in mouse tissues is increased. It can activate the target gene expression, such as nuclear factor Nrf2, and stimulate the cellular endogenous antioxidant system to decrease ROS levels [27]. In this study, we also revealed that the excessive increase in ROS stimulated the activation of the Nrf2/ARE antioxidant stress signalling pathway in the ovaries of the *Hmgcs2* KO mice (Fig. 6i). This may be the mechanism by which the primordial pool is decreased in the KO mice. However, the detailed molecular mechanism by which ketone body deficiency leads to a decreased follicle reserve needs to be explored. In addition, P3d ovary single-cell RNA sequencing showed that ketone body deficiency does not affect cell lineage development in the process of primordial follicle pool formation, which indicated that ketone body deficiency only causes excessive apoptosis of the follicle, but not the development of survived cells within the early ovary (Fig. 3e).

To confirm that primordial follicle pool formation is regulated by systemic ketone bodies rather than local ketone bodies in the ovaries, we also studied granulosa cell-specific *Hmgcs2* KO mice. Primordial follicle pool formation in the granulosa cell-specific *Hmgcs2* KO mice was not damaged (Fig. 4). Although we found that ketone bodies were at high levels in blood throughout the primordial follicle pool formation period, the local ketone body level produced by Hmgcs2 in granulosa cells is not clear. This will become an important research direction in the future.

In conclusion, we revealed that the nutrition of infants, especially ketone bodies in the newborns, plays an important role during the formation of the primordial follicle pool and affects the normal ovarian capacity. Our study provides a new metabolic explanation for POA and provides new possible preventive measures and treatment options for clinical POA patients.

## Materials and methods

### Animals

The C57BL/6J mice were obtained from GemPharmatech Co., Ltd (Jiangsu, China). Systemic *Hmgcs2* KO mice were generated by crossing *Hmgcs2*-floxed mice (*Hmgcs2*^*fl/fl*^) with EIIA-cre transgenic mice. Granulosa cell-specific *Hmgcs2* KO mice were generated by crossing *Hmgcs2*-floxed mice (*Hmgcs2*^*fl/fl*^) with AMHR2-cre transgenic mice. All mice were housed in groups in accordance with the regulations for mouse welfare and ethics of Nanjing University under strict conditions with controlled temperature and humidity under a 12-h dark–light cycle and free access to food and water. All animal experiments were carried out according to the Animal Care and Use Committee of the Model Animal Research Center of Nanjing University in Nanjing, China.

The P2d mice were treated with β-HB (400 mg/kg/day, 55188, Sigma) or mitoTEMO (1 mg/kg/day, SML0737, Sigma) through intraperitoneal injection, then were collected at P5d.

### Measurement of serum β-HB

The serum β-HB level was detected by a blook ketone tester (Keto Mojo). After the detector was calibrated, the detection band was inserted into the detector for use. A part of the tail tips were removed from mice in the CTL group and the KO group on different days, the outflow blood drops were added to the detection band, and the data were read and recorded. We measured 4–10 samples per group.

### Fertility assay

To assess mouse fertility, 6-week-old female *Hmgcs2-null* mice and CTL mice (wild-type or heterozygous) were mated with adult (8-week-old) C57BL/6J males of known fertility. The litter size and litter interval was recorded continuously. Two female mice mated with 1 male mouse, in total of 12 female mice.

### Vaginal cytology method

To detect the mice oestrus cycle, a vaginal swab was collected using a cotton tipped swab wetted with physiological saline and inserted into the vagina of the restrained mouse. The swab was gently turned and rolled against the vaginal wall and then removed. Cells were transferred to a dry glass slide by rolling the swab across the slide. The slide was air dried and then stained with Toluidine blue (Shanghai Macklin Biochemical) for 10 min. The slides were rinsed with water, dried in 37°C oven, and viewed immediately at 20 × magnification under bright field illumination. The stage of the oestrous cycle was determined based on the presence or absence of leukocytes, cornified epithelial, and nucleated epithelial cells according to Felicio *et al.* [28].

### Morphology, histology, and immunofluorescence

To assess the general morphology of *Hmgcs2-*null mice, mice were sacrificed at several developmental ages, and ovarian function and morphology were evaluated. Fresh tissues were fixed overnight at 4°C in 4% PBS-buffered paraformaldehyde for immunohistochemistry or Bouin’s fixative for HE staining, dehydrated in ethanol, embedded in paraffin, and cut into 5-μm sections continuously. For histological analysis, sections were subjected to HE staining according to the standard protocol. We counted the follicle number with 5 mice per group.

For immunofluorescence staining, the paraffin sections were dewaxed, rehydrated, and boiled in citrate buffer (pH 6.0) for antigen retrieval. Then, the sections were permeabilized with 0.1% Triton X-100, blocked with 10% goat serum and incubated with the indicated primary antibodies against MVH (Abcam, 1:200), TAp63 (Bioworld, 1:200), and γ-H2AX (Sigma, 1:200) overnight at 4°C. Afterwards, the slides were washed with PBS three times, incubated with secondary antibodies for 1–2 h at room temperature and then washed with PBS three times. A FluoView™ FV3000 confocal microscope (Olympus) was used to observe immunofluorescence staining.

### Follicle count

P21d mice ovary was embedded in paraffin and cut into 5-μm sections continuously, HE staining for one of every eight sections. The number of follicles was counted at different stages with visible oocyte nucleus under high power microscope, and the total number of different-stage follicles were multiplied by 8.

P1d, P3d, and P5d mice ovaries were embedded in paraffin and cut into 5-μm sections continuously, immunofluorescence staining of MVH for one of every six sections. The number of MVH-positive follicles was counted under A FluoView™ FV3000 confocal microscope (Olympus), and the total number of oocytes were multiplied by 6.

### TUNEL assay

To detect apoptosis, the *Hmgcs2-*null mice were sacrificed at several developmental ages. Fresh tissues were fixed overnight at 4°C in 4% PBS-buffered paraformaldehyde, dehydrated in ethanol, embedded in paraffin, and cut into 5-μm sections. We used a DeadEnd^TM^ colorimetric TUNEL assay (Promega). Sections were treated according to the assay instructions. A FluoView™ FV3000 confocal microscope (Olympus) was used to observe TUNEL staining. To measure the proportion of apoptotic follicles, we performed MVH immunofluorescence staining on sections adjacent to TUNEL staining sections. The ratio of TUNEL-positive follicle number to MVH-positive follicle number was ‘proportion of apoptotic follicles’.

### RNA isolation and real-time quantitative PCR (qPCR)

Mouse ovaries were carefully isolated and cleaned by removing any connective tissue. Total RNA was isolated from the mouse ovaries using TRIzol reagent (9109, Takara) according to the manufacturer’s instructions. Total RNA was measured with a NanoDrop 2000 (Thermo Fisher Scientific). Then, PrimeScript™ RT Master Mix (Takara) was used to reverse transcribe 1 μg RNA into cDNA according to the manufacturer’s instructions. Target gene and 18S primers were synthesized by Integrated DNA Technology (Invitrogen). qPCR was performed with ChamQ Universal SYBR qPCR Master Mix (Q711, Vazyme Biotech Co., Ltd) on the ViiA 7 Real-Time PCR System (Applied Biosystems). Each cDNA sample was run in triplicate, and target mRNA expression was normalized for RNA loading using 18S. The mRNA level in each sample was calculated relative to a vehicle control using the 2^−ΔΔCT^ analysis method.

### Western blot analysis

Total protein was extracted from frozen ovaries using NP40/RIPA buffer containing a protease/phosphatase inhibitor cocktail. The protein level of each sample was determined using the BCA Protein Assay (Bio-Rad Laboratories, Richmond, CA). Same amount of protein (50 μg) was separated by 10% SDS-PAGE, and resolved proteins were transferred to polyvinylidene fluoride membranes (Invitrogen) by standard protocols. Immunoblots were blocked with a 5% milk solution and incubated overnight at 4°C with primary antibodies against Hmgcs2 (Abcam, 1:1000) and α-tubulin (Proteintech, 1:1000). Goat antirabbit or antimouse secondary antibodies (1:5000) were used. Immunodetection was carried out using an enhanced chemiluminescence (ECL) kit.

### MDA assay

Mouse ovaries were carefully isolated and cleaned by removing any connective tissue. We used an MDA test kit (Solarbio) and detected the MDA level according to the instructions. 0.1 mL of PBS was added as a blank control in a centrifuge tube, 0.1 mL of different concentration standards were added for the preparation of the standard curve, and then 0.1 mL of the sample was added for the measurement, followed by the addition of 0.2 mL of the MDA test solution. Mixing and heating it in a boiling water bath for 15 min. The mixture was cooled to room temperature in a water bath and centrifuged at 4000 rpm for 10 min. The supernatant was taken and the absorbance was measured at 530 nm.

### Single-cell libraries and sequencing

Mouse P3d ovaries were carefully isolated and cleaned by removing any connective tissue. The Single Cell 3ʹ Protocol upgraded short read sequencers to deliver a scalable microfluidic platform for 3ʹ digital gene expression profiling of 500–10 000 individual cells per sample. The 10 × TM GemCodeTM Technology sampled a pool of ~750 000 barcodes to separately index each cell’s transcriptome. It did so by partitioning thousands of cells into nanoliter-scale Gel Bead-In-EMulsions (GEMs), where all generated cDNA shared a common 10× Barcode. Libraries were generated and sequenced from the cDNA and the 10 × Barcodes were used to associate individual reads back to the individual partitions.

Upon dissolution of the Single Cell 3ʹ Gel Bead in a GEM, primers containing (i) an Illumina® R1 sequence (read 1 sequencing primer), (ii) a 16 nt 10× Barcode, (iii) a 10 nt Unique Molecular Identifier (UMI), and (iv) a poly-dT primer sequence were released and mixed with cell lysate and Master Mix. Incubation of the GEMs then produced barcoded, full-length cDNA from poly-adenylated mRNA. After incubation, the GEMs were broken, and the pooled fractions were recovered.

Silane magnetic beads were used to remove leftover biochemical reagents and primers from the post GEM reaction mixture. Full-length, barcoded cDNA was then amplified by PCR to generate sufficient mass for library construction.

R1 (read 1 primer sequence) was added to the molecules during GEM incubation. P5, P7, the sample indexes, and R2 (read 2 primer sequences) were added during library construction via End Repair, A-tailing, Adaptor Ligation, and PCR. The final libraries containing the P5 and P7 primers were used in Illumina bridge amplification.

The Single Cell 3ʹ Protocol produced Illumina-ready sequencing libraries. A Single Cell 3ʹ Library comprised standard Illumina paired-end constructs which begined and ended with P5 and P7. The Single Cell 3ʹ 16 bp 10× Barcodes and 12 bp UMI were encoded in Read 1, while Read 2 was used to sequence the cDNA fragment. Sample index sequences were incorporated as the i7 index read. Read 1 and Read 2 were standard Illumina® sequencing primer sites used in paired-end sequencing.

### Statistical analysis

All data are presented as the mean±s.e.m. Statistical significance between two groups was analysed by an unpaired two-tailed Student’s t-test. Multiple groups were analysed by two-way ANOVA. *P< 0.05 was considered statistically significant.

## Supplementary Material

loac017_suppl_Supplementary_Figure

## Data Availability

All data are contained in the manuscript.
