# Supplementary material for: The neonatal ketone body is important for primordial follicle pool formation and regulates ovarian ageing in mice
Source: Life Metab. 2022 Aug 11;1(2):149–60. doi: 10.1093/lifemeta/loac017 (PMC11749118; doi:10.1093/lifemeta/loac017)
Supplement: loac017_suppl_Supplementary_Figure [file loac017_suppl_Supplementary_Figure.pdf]

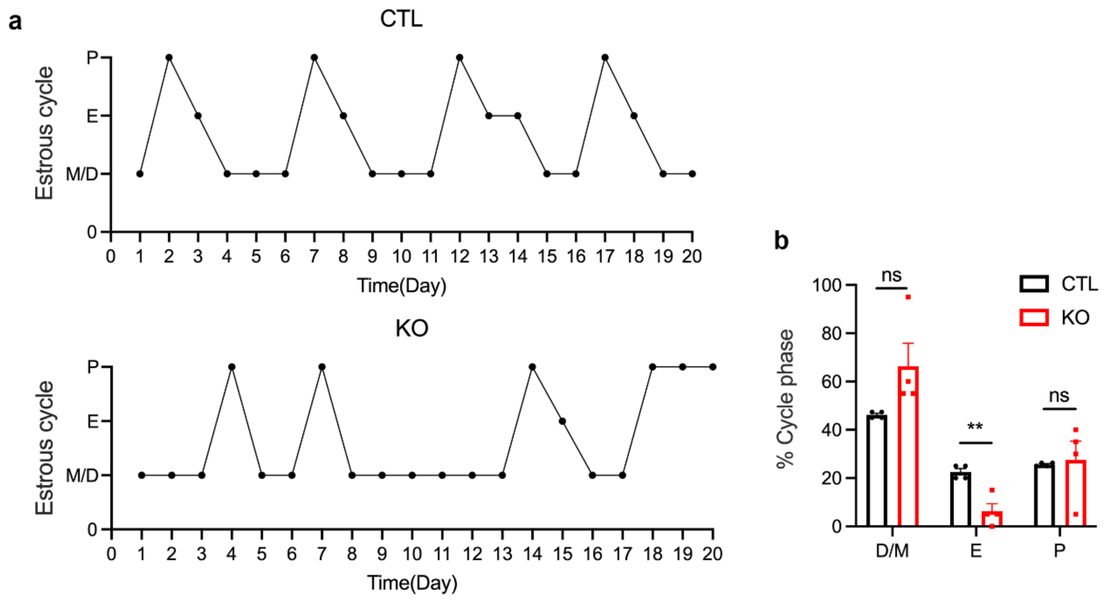

Supplementary Fig S1. The estrous cycle disorder in the 6-month-old Hmgcs2 KO mice. Data are shown as means ± s.e.m. $n = 4$, $^{**}P < 0.01$.
